# Transcriptome Reveals the Key Genes Related to the Metabolism of Volatile Sulfur-Containing Compounds in *Lentinula edodes* Mycelium

**DOI:** 10.3390/foods13142179

**Published:** 2024-07-10

**Authors:** Zheng Li, Fei Pan, Wen Huang, Shuangshuang Gao, Xi Feng, Meijie Chang, Lianfu Chen, Yinbing Bian, Wenli Tian, Ying Liu

**Affiliations:** 1 College of Food Science & Technology, Huazhong Agricultural University, Wuhan 430070, China; lizheng123@webmail.hzau.edu.cn (Z.L.); huangwen@mail.hzau.edu.cn (W.H.); 13720161459@163.com (S.G.); mjchang@webmail.hzau.edu.cn (M.C.); 2 State Key Laboratory of Resource Insects, Institute of Apicultural Research, Chinese Academy of Agricultural Sciences, Beijing 100193, China; yunitcon@yeah.net (F.P.); tianwenli@caas.cn (W.T.); 3 Department of Nutrition, Food Science and Packaging, San Jose State University, San Jose, CA 95192, USA; 4 Institute of Applied Mycology, Huazhong Agricultural University, Wuhan 430070, China; chenllianfu@foxmail.com (L.C.); bianyinbing@mail.hzau.edu.cn (Y.B.)

**Keywords:** *Lentinula edodes*, mycelium, sulfur compounds, RNA-seq, KEGG, molecular simulation

## Abstract

*Lentinula edodes* (*L. edodes*) is a globally popular edible mushroom because of its characteristic sulfur-containing flavor compounds. However, the formation of the volatile sulfur-containing compounds in the mycelium of *L. edodes* has not been studied. We found that there were also sulfur-containing aroma compounds in the mycelium of *L. edodes*, and the content and composition varied at different stages of mycelial growth and development. The γ-glutamyl-transpeptidase (GGT) and cysteine sulfoxide lyase (C-S lyase) related to the generation of sulfur compounds showed the highest activities in the 15-day sample. Candidate genes for the metabolism of volatile sulfur compounds in mycelium were screened using transcriptome analysis, including encoding the GGT enzyme, C-S lyase, fatty acid oxidase, HSP20, and P450 genes. The expression patterns of *Leggt*3 and *Leccsl*3 genes were consistent with the measured activities of GGT and C-S lyase during the cultivation of mycelium and molecular dynamics simulations showed that they could stably bind to the substrate. Our findings provide insights into the formation of sulfur-containing flavor compounds in *L. edodes*. The mycelium of *L. edodes* is suggested for use as material for the production of sulfur-containing flavor compounds.

## 1. Introduction

*Lentinula edodes* (*L. edodes*) is one of the most popular mushrooms with rich nutrients [1] and characteristic flavor [2]. Sulfur-containing compounds, mainly including thioethers, thiols, and sulfur-containing heterocyclic compounds, contribute to their characteristic flavor [3]. Among sulfide compounds, dimethyl sulfide, dimethyl trisulfide, and methanethiol have a higher content and a sulfur flavor, making them characteristic odor substances of *L. edodes* [4]. Sulfur-containing compounds are the main flavor components in dried *L. edodes*, and their formation is mainly influenced by the heating temperature and time [5,6]. Currently, it is widely believed that the formation of sulfur-containing flavor substances mainly goes through two stages. Firstly, γ-glutamyl-transpeptidase (GGT) catalyzes lentinic acid to γ-glutamy lentinic acid, and cysteine sulfoxide lyase (C-S lyase) catalyzes γ-glutamy lentinic acid to products such as thiosulfate. These products then undergo a series of non-enzymatic reactions, followed by condensation and cleavage, ultimately forming a series of sulfur-containing flavor compounds [7,8]. Our previous studies showed that these two enzymes were not only the key enzymes for the formation of sulfur-containing volatile compounds in *L. edodes* but also involved in the formation of endogenous formaldehyde, which is associated with food safety and human health [9,10]. The GGT and C-S lyase families of *L. edodes* have seven (*Leggt*1–7) and five (*Lecsl*1–5) genes, which use γ-glutamyl diaminopimelic acid and S-methyl-L-cysteine sulfoxide as substrates, respectively. In a previous study, we clarified the role of *Lecsl*2 in the formation of *L. edodes* flavor and tested the enzymatic activity of GGT at different stages of *L. edodes* [10,11]. However, the functions of other genes in the C-S lyase family and the specific catalytic mechanisms of GGT are still under investigation.

Research on the flavor of *L. edodes* mainly focused on the fruiting body stage, to understand the formation mechanisms of sulfur-containing flavor compounds in *L. edodes*. Researchers analyzed the differences in volatile components at different development stages of *L. edodes* fruiting bodies. The growth and development of *L. edodes* fruiting bodies take a long time and undergo physiological metabolism during storage and processing [8], thus it is difficult to use *L. edodes* fruiting bodies to produce sulfur-containing flavor compounds. Compared with the fruiting body, *L. edodes* mycelium has a shorter growth cycle, easier culture and regeneration, and easier genome-editing-related operations; thus, it is a better research object for revealing the formation mechanism of aroma components in *L. edodes* and also could be processed as a food-flavoring agent or additive [12,13,14,15]. Studies indicated that *L. edodes* mycelium also contains amino acids, fatty acids, polysaccharides, and other components beneficial to human health, while studies on mycelium mainly focused on fermentation to produce polysaccharides and other active components. Regarding the flavor of the mycelium, studies have shown that *L. edodes* mycelium contains non-volatile taste components [16,17,18,19,20,21]. However, it is unclear whether characteristic volatile substances can be affected by the growth and development of mycelium [22,23]. In order to make better use of *L. edodes* mycelium, it is necessary to understand its flavor formation. 

The objectives of this study were to explore the differences in flavor components in the prophase of *L. edodes* mycelial growth and screen candidate genes related to mycelium flavor formation and reveal the key gene action mechanism. Our findings will enable further studies of the formation mechanisms of *L. edodes* flavor and usage of *L. edodes* mycelium. 

## 2. Materials and Methods

### 2.1. Strain and Culture

*L. edodes*, strain W1 (ACCC 50926) was provided by the Institute of Applied Mycology, Huazhong Agricultural University. The strain W1 was cultured on MYG medium 2% malt extract (Shuangxuan company, Beijing, China), 2% glucose (Sinopharm Chemical Reagent Co., Ltd., Shanghai, China), 0.1% yeast extract (Angelyeast, Yichang, China), 0.1% peptone (Shuangxuan company, Beijing, China), 2% agar (Biofroxx, Heidelberg, Germany) in 9 cm plates for 15, 35, and 55 days, respectively.

### 2.2. Microstructure Analysis by Scanning Electron Microscope (SEM)

The mycelium was transferred into 2.5% glutaraldehyde solution and fixed for 4 h. After the mixture was dried and gold sprayed, it was viewed under SEM to observe the microstructure. The magnification of the scanning electron microscope was 500.

### 2.3. Flavor Extraction and Analysis

2-butanone, 2-pentanone, 2-hexanone, 2-heptanone, 2-octanone, 2-nonone mixed standard (1 mg/L) used for analysis was purchased from German G.A.S (Hong Kong, China). One gram of dried mycelium was used to determine the volatile components by gas chromatography–ion mobility spectroscopy (GC-IMS). The headspace sampling conditions were as follows: incubation 10 min at 60 °C and sampling probe temperature 85 °C. GC-IMS conditions were as follows: MXT-5 chromatographic column (15 m × 0.53 mm, 1.0 μm), column temperature 60 °C, analysis time 30 min, IMS temperature 45 °C, and drift gas flow rate 150 mL/min. The carrier gas flow rate was 2 mL/min for 2 min and increased to 10 mL/min across the next 8 min, then increased to 100 mL/min across the next 15 min, and maintained there for an additional 5 min. A GC-IMS instrument supporting analysis software Vocal (version 0.1.3) was used for information collection and analysis. The application software had built-in Reporter and Gallery Plot plug-ins to plot the two-dimensional and fingerprint chromatograms of volatile components in the samples. C4~C9 ketones were used as the external standard standards to calculate the retention index of volatile components, which were used to match with the built-in National Institute of Standards and Technology (NIST) and IMS databases to conduct qualitative analysis of volatile components.

### 2.4. Enzyme Activities

The activities of GGT and C-S lyase were determined using our previous methods [10]. γ-glutamyl p-nitroaniline was used as a substrate to determine the GGT activity. S-methyl-L-cysteinyl was used as a substrate to measure the C-S lyase activity. Specific enzyme activity (U/g) was defined as the production of 1μ Mol p-nitroaniline or pyruvate per gram of total mycelium per minute.

### 2.5. RNA-seq and Transcriptomes Analysis

The RNA-seq sequencing experiment process included sample detection, library construction, quality inspection, and computer sequencing. The Illumina NovaSeq6000 sequencing platform (San Diego, CA, USA) was used for sequencing in PE150 mode. The original data were filtered to obtain clean data, and the sequence was aligned with the previously published reference genome of *L. edodes* strain W1-26 [24]. We used three different methods, edgeR, DESeq2, and log2FC, to calculate the number of differentially expressed genes. The intersection of the results from these three methods was taken. For edgeR and DESeq2, the threshold was *p* ≤ 0.001. For log2FC, the threshold was set to an absolute value greater than or equal to 2, which means a fold change of at least 4.

### 2.6. Transcriptome Validation

Due to individual differences among samples, in order to verify the reliability of transcriptome results, quantitative real-time PCR was used to measure the expression of related genes in samples with the same treatment but without transcriptome. The total RNA was extracted and reverse-transcribed to cDNA by HiScript II Q RT SuperMix for Qpcr (+gDNA wiper). Next, the AceQ qPCR SYBR Green Master Mix was used to detect the relative expression levels. Post-qRT-PCR calculations of relative gene expression levels were performed according to the method described in [25]. The primers for qRT-PCR were designed by Primer 3 (https://www.bioinformatics.nl/cgi-bin/primer3plus/primer3plus.cgi, accessed on 7 July 2024).

### 2.7. Molecular Docking and Molecular Dynamic Simulation

The 3D structures of GGT and CSL proteins were constructed using AlphaFold2 [26], with Global plDDT values of 91.699 and 92.671, respectively, indicating the reliability of the generated structures. The structures of S-methyl-L-cysteine sulfoxide and p-nitroaniline were built using the Avogadro program [27], and energy minimization was performed using the MMFF94s force field to optimize bond angles and lengths. Subsequently, semi-flexible molecular docking was carried out using the Autodock Vina [28] program. For the docking process, the docking coordinates for the Leggt3 protein were set as x = −1.8, y = 10.6, and z = 1.4, while for the Lecsl3 protein, the coordinates were x = −11.8, y = 2.8, and z = −9.6. The exhaustiveness parameter was set to 50, which determines the thoroughness of the docking search. During docking, the protein structure remained rigid while allowing the ligand’s rotatable bonds to move. After the docking process, the optimal complex construction was selected for molecular dynamics (MD) simulation study based on the binding energy and binding pose.

Based on molecular docking, molecular dynamics simulations were performed on GGT-γ-glutamyl p-nitroaniline and CSL-S-methyl-L cysteine sulfoxide. The geometric optimization of p-nitroaniline and methyl-L-cysteine sulfoxide was carried out using the ORCA [29] program with the r2SCAN-3c functional, and their single point energies were calculated using B3LYP/G D3 def2-TZVP def2/J RIJCOSX. The topological information of nitroaniline and S-methyl-L cysteine sulfoxide was generated using Ambertools [30] and their RESP charges were fitted using the Multiwfn program [31]. The systems were placed in a cubic TIP3P water tank and counter ions were added to neutralize the total charge of the system, maintaining a neutral system. Energy minimization was performed using the steepest descent method (1000.0 kJ/mol/nm) followed by conjugate gradient optimization (100.0 kJ/mol/nm) for the second energy minimization. Subsequently, the system was pre-equilibrated using NVT (1 ns, 298 K) and NPT (1 ns, 1 Bar) simulations, respectively.

### 2.8. Statistical Analysis

The experiments were conducted in triplicate and values were expressed as means ± SD (standard deviation). Data were analyzed using GraphPad Prism 5. The KEGG analysis was plotted using bioinformatics (https://www.bioinformatics.com.cn) [32]. The volcano map was drawn using Image GP (https://www.bic.ac.cn/ImageGP/, accessed on 7 July 2024) [33]. 

## 3. Results

### 3.1. Morphology and Microstructure Analysis of Mycelium

**Figure 1:**
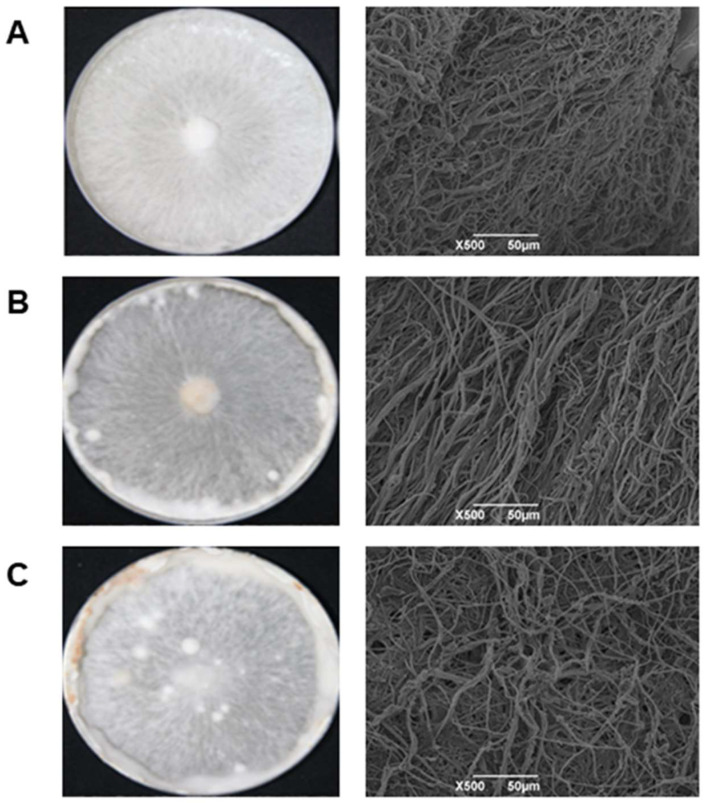
Macro morphology and microstructure of mycelium cultured for 15 days (**A**), 35 days (**B**), and 55 days (**C**), respectively.

The photographs of *L. edodes* mycelium collected at three different developmental stages are shown in Figure 1. As can be seen, the morphology of *L. edodes* mycelium changed significantly with the cultivated time. After 15 days of culturing, the mycelium extended to the edge of the culture dish. At this period, the mycelium was relatively dense, which was illustrated in the SEM results. As the space in the culture dish was limited, the mycelium began to differentiate [34]. When the culture time reached 35 days, the mycelium became denser. After 55 days of cultivation, the mycelium aggregated intensively and caused a significant increase in the number of kinks in the culture dish, and the mycelium at the edge turned brown [35,36]. During the early development stages of *L. edodes* mycelium, its biomass gradually increases and accumulates.

### 3.2. Analysis of Volatile Components in Mycelium

**Figure 2:**
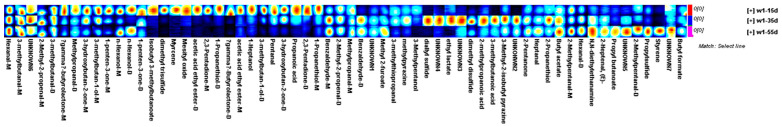
Fingerprint of the main volatile components in mycelium cultured for different numbers of days.

In this experiment, we detected volatile components in *L. edodes* at different mycelium stages through GC-IMS. As shown in Figure 2, the volatile components of the mycelium mainly varied with the culturing time. The main volatile components of *L. edodes* mycelium were acids, aldehydes, alcohols, and ketones, which were produced through amino acid, fatty acid, and carbohydrate metabolisms [37]. The levels of hexanal-M, 3-methylbutanal-M, 2-Methyl-2-propenal-M, and 3-methylbutanal-D did not change over time, indicating that they might be stable components of the *L. edodes* mycelium. *L. edodes* mycelium cultured for 15 and 55 days contained a higher level of n-Hexanol than that cultured for 35 days. The sulfur-containing volatile compounds in the mycelium cultured for 15 days were dimethyl trisulfide and 1-propanethiol. Dimethyl disulfide, diallyl sulfide, and 2-propanethiol were detected in the mycelium cultured for 35 and propylsulfide was detected in the mycelium cultured for 55 days [38,39]. Our results indicated that sulfur-containing volatile compounds in *L. edodes* were generated at the mycelium stage as well as hexanal-M, 3-methylbutanal-M, 2-methyl-2-propenal-M, and 3-methylbutanal-D. In summary, the *L. edodes* mycelium cultured for 15 days had the highest levels of total volatile components and sulfur-containing volatile compounds, possibly because of the vitality of *L. edodes* mycelium. 

### 3.3. Enzyme Activities Analysis

**Figure 3:**
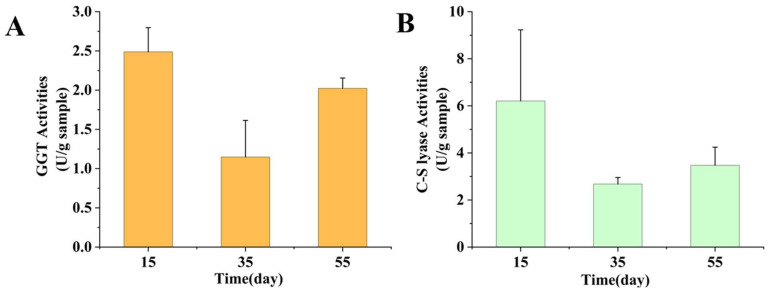
Activities of GGT (**A**) and C-S lyase (**B**) in mycelium cultured for different numbers of days.

The GGT and C-S lyase have been reported to be the key enzymes in the formation of volatile compounds in *L. edodes*; to reveal the mechanism of flavor formation in *L. edodes* mycelium, their activities in mycelium cultured for 15, 35, and 55 days were measured. The GGT and C-S lyase activities in different mycelium samples are shown in Figure 3. The GGT and C-S lyase activities of the 15-day sample were much higher than those of the 35- and 55-day samples, which could be why more volatile components were produced in the mycelium after 15 days. The GGT and C-S lyase activities of the 35-day sample were the lowest in the present study, and it was clear from the GC-IMS that this sample contained only a little n-Hexanol.

### 3.4. RNA-seq and Transcriptomes Analysis

**Figure 4:**
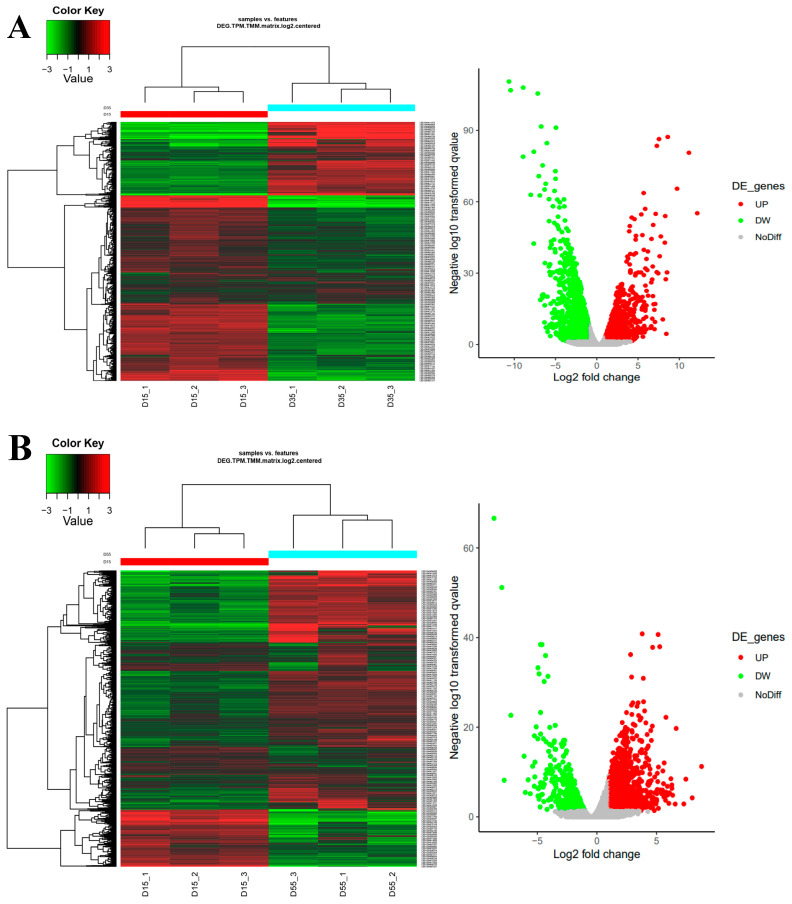

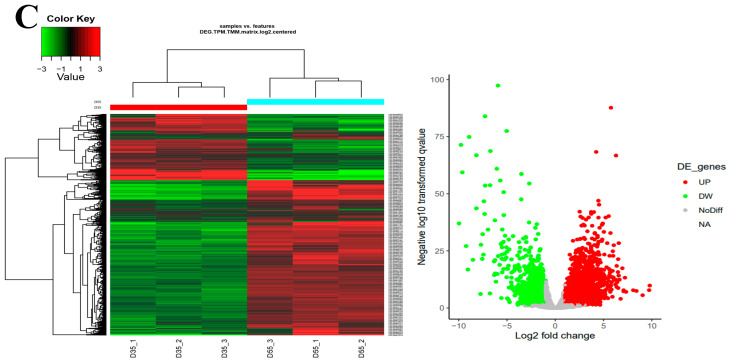
Differentially expressed genes (DEGs) in mycelium cultured for different days. D15, D35, and D55 represent *L. edodes* mycelium cultured for 15, 35, and 55 days, respectively.On the left is the heat map of the differential expression genes. This figure reflects the differential genes and gene expression changes in pairwise comparison of samples. For the color key, green means downregulation, and red represents upregulation of the transcripts. On the right is the volcano plot of DEGs in pairwise comparison of samples. This displays the analysis results of differentially expressed genes; in the figure, the horizontal axis represents the logarithmic value of the difference multiple, and the vertical axis represents the logarithmic value of the q value. Red is used to distinguish significantly upregulated genes, green is used to distinguish significantly downregulated genes (**A**) D15 vs. D35, (**B**) D15 vs. D55, (**C**) D35 vs. D55.

In order to screen for differentially expressed genes with significant changes in expression levels, we used three different methods: edgeR, DESeq2, and log2FC (Figure 4). Comparing 35-day mycelium with 15-day mycelium, edgeR, DESeq2, and log2FC identified 659, 997, and 489 upregulated differentially expressed genes, respectively. Across all methods, there were 376 genes consistently identified as upregulated differentially expressed genes. For downregulated differentially expressed genes, edgeR, DESeq2, and log2FC identified 1378, 5650, and 735 genes, respectively. There were 525 genes consistently identified as downregulated differentially expressed genes across all methods. Comparing 55-day mycelium with 15-day mycelium, edgeR, DESeq2, and log2FC identified 1157, 1241, and 699 upregulated differentially expressed genes, respectively. Among these methods, there were 421 genes consistently identified as upregulated differentially expressed genes. For downregulated differentially expressed genes, edgeR, DESeq2, and log2FC identified 835, 4660, and 258 genes, respectively. Across all methods, there were 170 genes consistently identified as downregulated differentially expressed genes. Comparing 55-day mycelium with 35-day mycelium, edgeR, DESeq2, and log2FC identified 2097, 2065, and 1351 upregulated differentially expressed genes, respectively. Among these methods, there were 962 genes consistently identified as upregulated differentially expressed genes. For all differentially expressed genes, edgeR, DESeq2, and log2FC identified 1548, 1754, and 410 downregulated genes, respectively. Among these methods, there were 226 genes consistently identified as downregulated differentially expressed genes.

**Figure 5:**
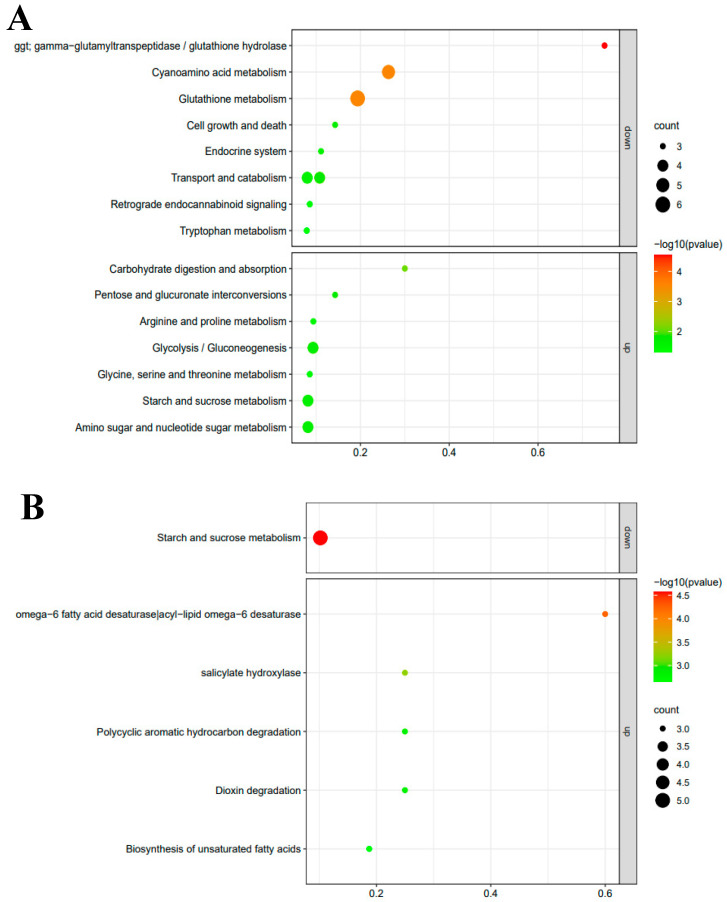

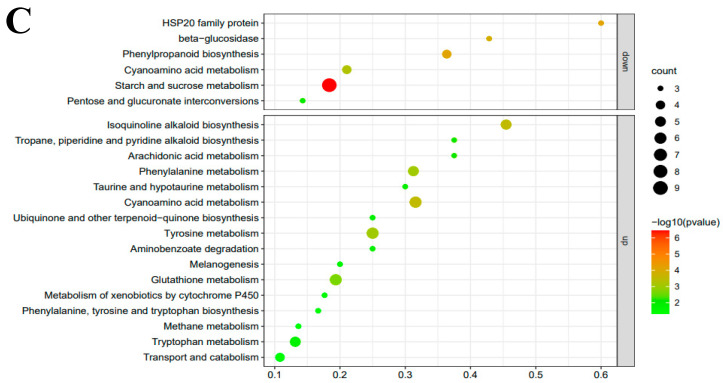
Kyoto Encyclopaedia of Genes and Genomes (KEGG) enrichment analysis results. KEGG enrichment analysis of genes and metabolic pathways involved in significant downregulation and upregulation of expression levels between different samples. D15, D35, and D55 represent *L. edodes* mycelium cultured for 15, 35, and 55 days, respectively. (**A**) D15 vs. D35; (**B**) D15 vs. D55; (**C**) D35 vs. D55.

In order to screen candidate genes related to the metabolisms of sulfur-containing volatile compounds, KEGG enrichment analysis was conducted on the genes involved in the metabolic pathways. The metabolic pathways with significant changes are shown in Figure 5. Compared with the 15-day sample, three genes on the GGT-related metabolic pathway, *Leggt*2 (LE01Gene02907), *Leggt*3 (LE01Gene03045), and *Leggt*6 (LE01Gene06030), decreased significantly in the 35-day sample. The expressions of six genes in the glutathione metabolic pathway were also downregulated in the 35-day sample. Interestingly, four of the six genes belonged to the GGT gene family—the three mentioned above and *Leggt*4 (LE01Gene05244). However, the four GGT family genes related to the glutathione metabolism pathway were upregulated in the 55-day sample compared to the 35-day sample (Figure 5C). The results showed that the expression levels of the four GGT genes decreased first and then increased in mycelium during 55 days’ growth, which was consistent with the changes in GGT activity. Our results indicated that the changes in the expression of Leggt2–4 and Leggt6 genes directly affected the activity of GGT in the mycelium. However, there were few metabolic pathways and genes with significant changes in the 55-day sample compared with the 15-day sample. The differences were found in fatty acid and carbohydrate metabolisms (starch and sucrose metabolism). The genes enriched in two fatty acid metabolism pathways were LE01Gene02815, LE01Gene02816, and LE01Gene02817. The three genes encoded the fatty acid dehydrogenase (FAD) were reported to produce varied volatile profiles in *L. edodes* at the different fruiting body stages. In summary, the annotated metabolic pathways in mycelium were mainly amino acid, carbohydrate metabolic pathways, cyanoamino acid metabolism, glycolysis/gluconeogenesis, tryptophan metabolism, pentose and glucuronate interconversions, taurine and hypotaurine metabolism, arginine and proline metabolism and arachidonic acid metabolism. These results were similar to the metabolic pathways annotated in the fruiting bodies [40]. KEGG enrichment results showed that these candidate genes participate in flavor formation in *L. edodes* at the mycelium stage. Results suggested that the genes of the GGT family, FAD, HSP20, and P450 could be involved in the metabolism of sulfur-containing volatile compounds in *L. edodes*.

### 3.5. Transcriptome Validation

**Figure 6:**
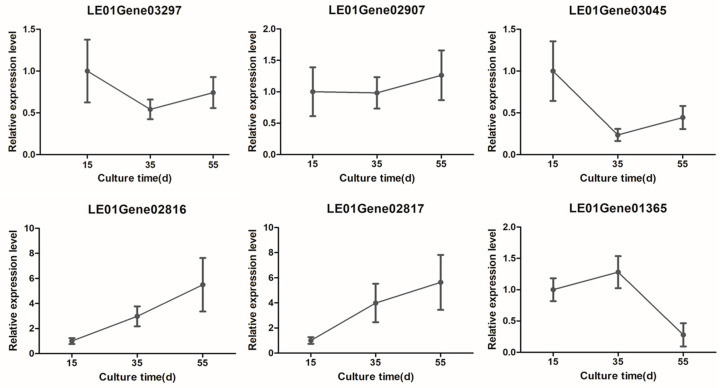
Verification of the expression levels of selected transcripts by qRT-PCR.

In order to verify the reliability of the transcriptome results, the expression of related genes was measured with the same treatment but without transcriptome sequencing [41]. As shown in Figure 6, the expression levels of the LE01Gene03297, LE01Gene02907, LE01Gene03045, LE01Gene02816, LE01Gene02817, and LE01Gene01365 genes were consistent with the results of the transcriptome analysis. The expression patterns of the *Leggt*3 (LE01Gene03045), and *Leccsl*3 (LE01Gene03297) genes were consistent with the measured activities of GGT and C-S lyase during the cultivation of the mycelium.

### 3.6. Mechanisms of Leggt3 and Lecsl3

**Figure 7:**
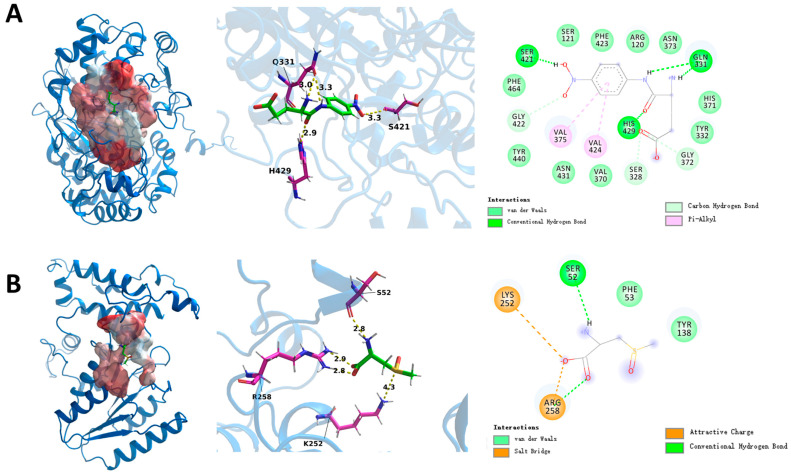
From left to right are: the region and overall conformation of protein substrate binding; the local conformation, binding sites, and bond lengths of protein substrate binding region; the binding sites and binding modes of protein substrate binding. (**A**) *Leggt*3 with γ-glutamyl p-nitroaniline. (**B**) *Lecsl*3 with S-methyl-L cysteine sulfoxide.

The results of RNA-seq and qRT-PCR revealed that Leggt3 and Lecsl3 seemed to be the essential enzymes in the GGT and C-S lyase family, thus we chose to reveal the mechanisms of their interaction with substrates. When cloning the Lecsl3 gene, we discovered the actual length of the coding region was 966 bp instead of the 1497 bp shown in the database. The mutation of the 966th base from A to G made the 322nd amino acid code become the terminator TGA, which caused an early termination of protein translation. Therefore, we modeled the protein using the obtained sequence. Through molecular docking, as shown in Figure 7, we found that the substrates can bind to the hydrophobic pockets of *Leggt*3 and *Lecsl*3 and interact with key residues. *Leggt*3 mainly forms hydrogen bonds and Pi-Alkyl with the substrate, and the amino acid binding sites involved in substrate binding are VAL-375, VAL-424, GLN-331, HIS-429, and SER-421. In addition, there were some amino acids around the substrate that interact with the substrate through carbon-hydrogen bonds (Figure 7A). *Lecsl*3 forms hydrogen bonds and salt bridges with the substrate, LYS-252 forms hydrogen bonds with the substrate, and SER-52 forms salt bridges with the substrate, while AGR-258 forms salt bridges with the oxygen anion and hydrogen bonds with the oxygen atom (Figure 7B).

**Figure 8:**
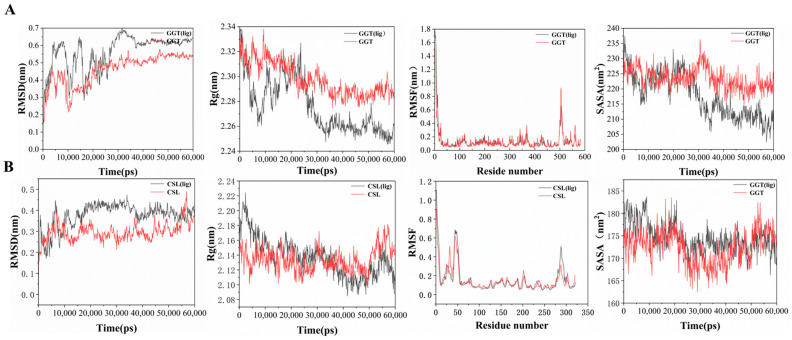
Root-mean-square deviation (RMSD), radius of gyration (Rg), root-mean-square fluctuation (RMSF), and solvent-accessible surface area (SASA) before and after protein substrate binding. (**A**) *Leggt*3, (**B**) *Lecsl*3.

To investigate the stability of protein–substrate binding and the impact on the protein after binding, we performed a 60 ns molecular dynamics simulation based on the docking results, as shown in Figure 8, comparing the protein conformational changes between binding and non-binding of substrates. Calculation of the root-mean-square deviation (RMSD) of the protein backbone can assess the stability of protein conformation [42]. The radius of gyration (Rg) can reflect the potential plasticity of the protein structure [43]. As shown in Figure 8, according to the results of the RMSD analysis, the two systems gradually converged during the simulation process. Compared to the substrate-free *Leggt*3, the RMSD value of *Leggt*3 increases upon substrate binding, indicating that substrate binding induces conformational changes in *Leggt*3 and promotes the formation of an active site with catalytic functionality. *Lecsl*3 also exhibits a similar phenomenon upon substrate binding, but of smaller magnitude, reaching convergence within a very short time (~20 ns). The change in Rg value before and after protein–substrate binding better elucidates this phenomenon; this indicates that the protein structure becomes more compact after substrate binding. Root-mean-square fluctuation (RMSF) is commonly used to calculate the fluctuation in individual residues, thereby indicating the degrees of freedom of residue movements. We evaluated the flexibility of each amino acid residue in the protein before and after binding to the substrate by calculating the RMSF values. The results revealed that for both proteins, the RMSF values of most amino acids did not fluctuate before and after substrate binding, except for a few amino acids that displayed minor fluctuations.

Due to the molecular dynamics simulation being carried out in an aqueous environment, we also calculated the solvent-accessible surface area (SASA) (Figure 8) [44,45] of *Leggt*3 and *Lecsl*3 before and after binding with the substrate. From the graph (Figure 8), we can observe that the SASA value of *Leggt*3 decreased and its hydrophobicity was enhanced after binding with the substrate. In contrast, the SASA of *Lecsl*3 showed little change before and after substrate binding, and the SASA values were lower than those of *Leggt*3. This may be because the active pocket of *Leggt*3 was exposed and it interacts more strongly with water when the substrate binds. Conversely, the active pocket of *Lecsl*3 was smaller and partially exposed; therefore, even after substrate binding, there was no significant change in its contact with water.

## 4. Discussion

This study revealed the rule of flavor formation of *L. edodes* mycelium at different growth stages, enriching research on the flavor of *L. edodes* at various growth stages. By observing the growth of the mycelium, the biomass of mycelium was found to increase in the early stage of growth, and the hyphae gathered into clusters. Several sulfur-containing volatile compounds (dimethyl trisulfide, dimethyl disulfide, propylsulfide, and propanethiol) were detected in *L. edodes* mycelium. However, mycelium cultured for different numbers of days showed various levels of sulfur-containing volatile compounds. These results indicate that the sulfur-containing characteristic flavor components of *L. edodes* are already produced at the mycelium stage. In order to confirm the origins of sulfur-containing flavor components in mycelium, activities of GGT and C-S lyase were investigated, The results indicated that the enzyme activities of GGT and C-S lyase have a certain impact on the composition and content of aroma compounds.

By comparing transcriptome and KEGG analyses with the research results on the flavor components of *L. edodes* fruiting bodies at different stages, we also found that genes expect HSP20 and P450, which affect the growth and flavor differences of *L. edodes* mycelium and the fruiting bodies, are almost identical in the mycelium and fruiting bodies. This indicates that the influence of these genes on the flavor of *L. edodes* persists throughout. HSP20 is the ubiquitous heat shock protein in organisms and plays an important role in the heat tolerance of organisms, and sulfur-containing volatile compounds in *L. edodes* are generated by heat. It seemed that there might be a correlation between HSP20 and the generation of sulfur-containing volatile compounds, but there was not sufficient evidence to be certain. A study reported that the overexpression of HSP20 could promote the growth of *L. edodes* mycelium [46]. Therefore, this implies that HSP20 might affect the growth of *L. edodes* mycelium. Like HSP20, P450 is a ubiquitous enzyme system in organisms, and it has functional roles in growth, development, and secondary metabolisms [47,48]. Previous studies proved that P450 was vital during the production of sulfur-containing volatile compounds in plants [49,50]. In the present study, several P450 family genes were found to be related to the formation of sulfur-containing volatile compounds in the mycelium, including LE01Gene03374, LE01Gene04094, and LE01Gene10823. Elucidating the functions of HSP20 and P450 genes in the generation mechanism of volatile sulfur compounds in *L. edodes* is envisaged in the future. During the process of transcriptome verification, we found that the variation pattern of *Leggt*3 and *Lecsl*3’s gene expression was consistent with the trend of activity changes of GGT and C-S lyase. The potential binding sites and interactions with the substrate were determined through molecular docking calculations. Based on the molecular docking results, molecular dynamics simulations showed that they can stably bind to the substrate. However, as proteins in eukaryotic organisms, revealing whether they function as oligomers or require post-translational modifications to become active needs further experiments.

In this study, we systematically studied the formation mechanism of flavor in *L. edodes* mycelium and conducted a brief functional analysis of key genes. The growth of *L. edodes* from mycelium to fruiting bodies involves several stages: mycelium colonization in the cultivation bag, mycelium color transformation, primordium formation, and fruiting body emergence. It takes about two months from mycelium inoculation in the cultivation bag to complete mycelium color transformation. Therefore, we focused on the flavor formation mechanism in *L. edodes* mycelium before complete color transformation. Compared to fruiting bodies, mycelium is easier to obtain and cultivate in large quantities. These results supported the hypothesis that *L. edodes* mycelium can be studied to explore the mechanisms underlying flavor formation and it is possible to use the mycelium to produce characteristic *L. edodes* flavor. We identified some genes that may affect the flavor of *L. edodes* mycelium, and the formation of relevant flavor components of *L. edodes* can be regulated by controlling the expression of these genes.

## 5. Conclusions

The present study revealed the characteristics of mycelial growth and flavor formation in the mycelium stage of *L. edodes*, and a preliminary investigation of the mechanism of action of *Leggt*3 and *Lecsl*3 has also been conducted. In the first two months of growth, the amount of *L. edodes* mycelium continues to increase and the hyphae gather into clusters. During this process, characteristic *L. edodes* flavor compounds dimethyl trisulfide and dimethyl disulfide were also formed, and the genes and metabolic pathways related to flavor and the growth of *L. edodes* were the same in both the mycelium and fruiting body stages. The results not only provide novel insights for the study of sulfur-containing flavor compounds and formaldehyde biosynthesis in *L. edodes* but also lay a foundation for utilizing mycelium fermentation to produce sulfur-containing flavor compounds.

## Data Availability

The original contributions presented in the study are included in the article, further inquiries can be directed to the corresponding author.
